# Childhood Trauma and Social Cognition Deficits in Early Psychosis: Evidence from the PREGAP Study

**DOI:** 10.1192/j.eurpsy.2026.10994

**Published:** 2026-07-31

**Authors:** B. Pedruzo, C. Aymerich, L. Madaria, G. Acasuso, M. Á. Gonzalez-Torres, A. Catalán

**Affiliations:** 1 Psychiatry Department, Basurto University Hospital, Bilbao, Spain; 2 Child and Adolescent Psychiatry, King’s College London, London, United Kingdom; 3 Biobizkaia Health Research Institute, Barakaldo, Spain

## Abstract

**Introduction:**

Adverse childhood experiences (ACEs) are disproportionately prevalent in early psychosis and may contribute to impairments in social cognition, a core predictor of functional outcomes. However, it remains unclear whether trauma explains unique variance in social cognition beyond the effects of psychosis itself.

**Objectives:**

To examine whether ACE exposure contributes independently to deficits in social cognition across individuals with first-episode psychosis (FEP), clinical high risk for psychosis (CHR-P), and healthy controls (HC).

**Methods:**

In this cross-sectional study, 107 participants were included (39 FEP, 35 CHR-P, 33 HC). ACEs were assessed with the Childhood Trauma Questionnaire (CTQ). Social cognition was measured using a comprehensive battery covering theory of mind (MASC, Hinting, Faux Pas), facial emotion recognition (PERE), and general socio-cognitive functioning (GEOPTE). Linear regression models adjusted for age, sex, and IQ were performed. Three nested models were compared: Model A (ACE), Model B (clinical group), and Model C (combined). Interaction terms tested stage-dependent effects.

**Results:**

Higher ACE exposure was significantly associated with worse performance in specific social cognition domains. Emotional abuse predicted greater socio-cognitive difficulties (GEOPTE; β=0.62, p=0.01), physical and sexual abuse were linked to impaired disgust recognition (β=-0.06 and -0.05, both p=0.02), and emotional neglect was related to reduced Hinting performance (β=-0.11, p=0.02) and recognition of neutral expressions (β=-0.09, p<0.01). Physical neglect was associated with both reduced recognition of neutrality (β=-0.14, p<0.01) and increased recognition of joy (β=0.06, p=0.05). Nested model comparisons showed that adding ACEs improved model fit beyond clinical status, particularly for neutral expressions (ΔR^2^ up to 0.18, p<0.01). ACE-by-group interactions indicated that negative effects of abuse on disgust recognition were stronger in FEP and CHR-P than in HC.

**Image 1:**
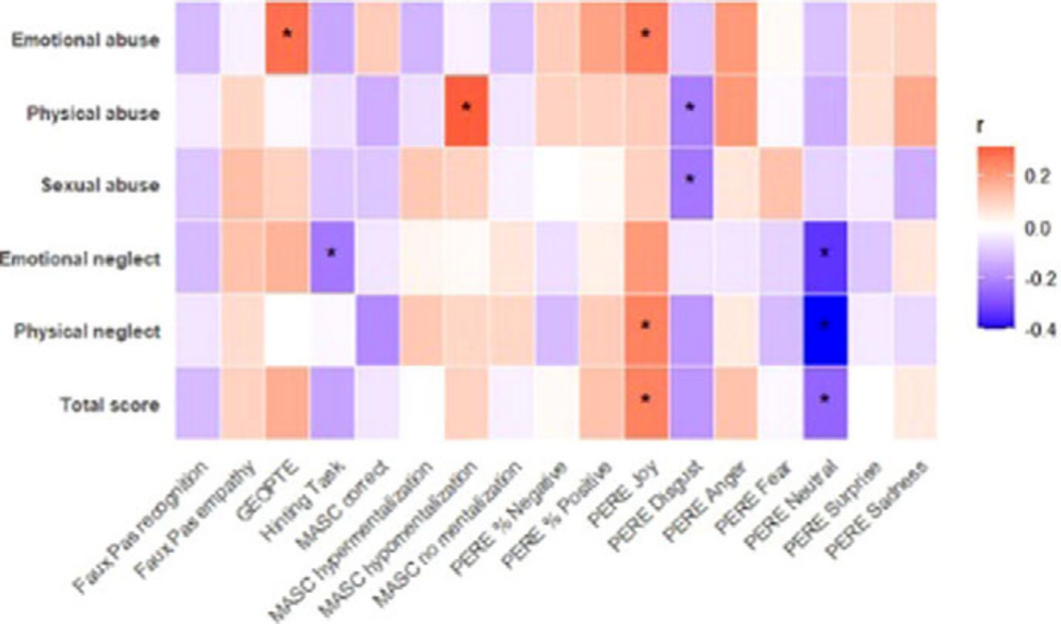
Image 1: Long description.

**Image 2:**
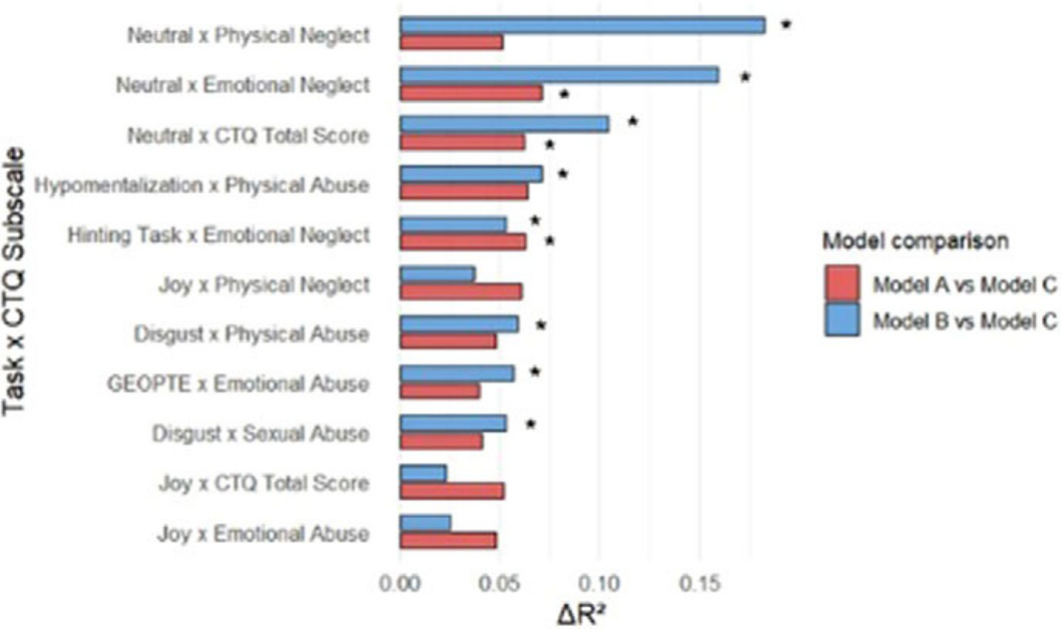
Image 2: Long description.

**Conclusions:**

Childhood trauma contributes independently and additively to deficits in theory of mind and emotion recognition in early psychosis. Findings underscore the need to integrate trauma-informed approaches in assessment and intervention for CHR-P and FEP populations.

**Disclosure of Interest:**

None Declared

## Long descriptions

### Image 1: Long description

A heatmap displays the correlation coefficient r on a color scale from dark blue (negative 0.4) to white (0.0) to dark orange (positive 0.2).

The Y-axis lists six categories from top to bottom: Emotional abuse, Physical abuse, Sexual abuse, Emotional neglect, Physical neglect, and Total score.

The X-axis lists 15 tasks from left to right: Faux Pas recognition, Faux Pas empathy, G E O P T E, Hinting Task, M A S C correct, M A S C hypermentalization, M A S C hypomentalization, M A S C no mentalization, P E R E percent Negative, P E R E percent Positive, P E R E Joy, P E R E Disgust, P E R E Anger, P E R E Fear, P E R E Neutral, P E R E Surprise, and P E R E Sadness.

Significant correlations marked with an asterisk include:

* Emotional abuse: Positive correlation with G E O P T E and P E R E Joy.

* Physical abuse: Positive correlation with M A S C hypomentalization and negative correlation with P E R E Disgust.

* Sexual abuse: Negative correlation with P E R E Disgust.

* Emotional neglect: Negative correlation with Hinting Task and P E R E Neutral.

* Physical neglect: Positive correlation with P E R E Joy and negative correlation with P E R E Neutral.

* Total score: Positive correlation with P E R E Joy and negative correlation with P E R E Neutral.

Navigate back to Image 1:.

### Image 2: Long description

The horizontal bar chart plots delta R super 2 on the x-axis, ranging from 0.00 to 0.15, against Task x C T Q Subscale on the y-axis. A legend on the right identifies red bars as Model A vs Model C and blue bars as Model B vs Model C. Asterisks indicate statistical significance.

From top to bottom, the interactions are:

* Neutral x Physical Neglect: Model B vs C is highest at approximately 0.18 with an asterisk. Model A vs C is approximately 0.05.

* Neutral x Emotional Neglect: Model B vs C is approximately 0.16 with an asterisk. Model A vs C is approximately 0.07.

* Neutral x C T Q Total Score: Model B vs C is approximately 0.11 with an asterisk. Model A vs C is approximately 0.06 with an asterisk.

* Hypomentalization x Physical Abuse: Model B vs C is approximately 0.07 with an asterisk. Model A vs C is approximately 0.06.

* Hinting Task x Emotional Neglect: Model B vs C is approximately 0.05 with an asterisk. Model A vs C is approximately 0.06 with an asterisk.

* Joy x Physical Neglect: Model B vs C is approximately 0.04. Model A vs C is approximately 0.06.

* Disgust x Physical Abuse: Model B vs C is approximately 0.06 with an asterisk. Model A vs C is approximately 0.05.

* G E O P T E x Emotional Abuse: Model B vs C is approximately 0.06 with an asterisk. Model A vs C is approximately 0.04.

* Disgust x Sexual Abuse: Model B vs C is approximately 0.05 with an asterisk. Model A vs C is approximately 0.04.

* Joy x C T Q Total Score: Model B vs C is approximately 0.02. Model A vs C is approximately 0.05.

* Joy x Emotional Abuse: Model B vs C is approximately 0.02. Model A vs C is approximately 0.05.

Navigate back to Image 2:.

